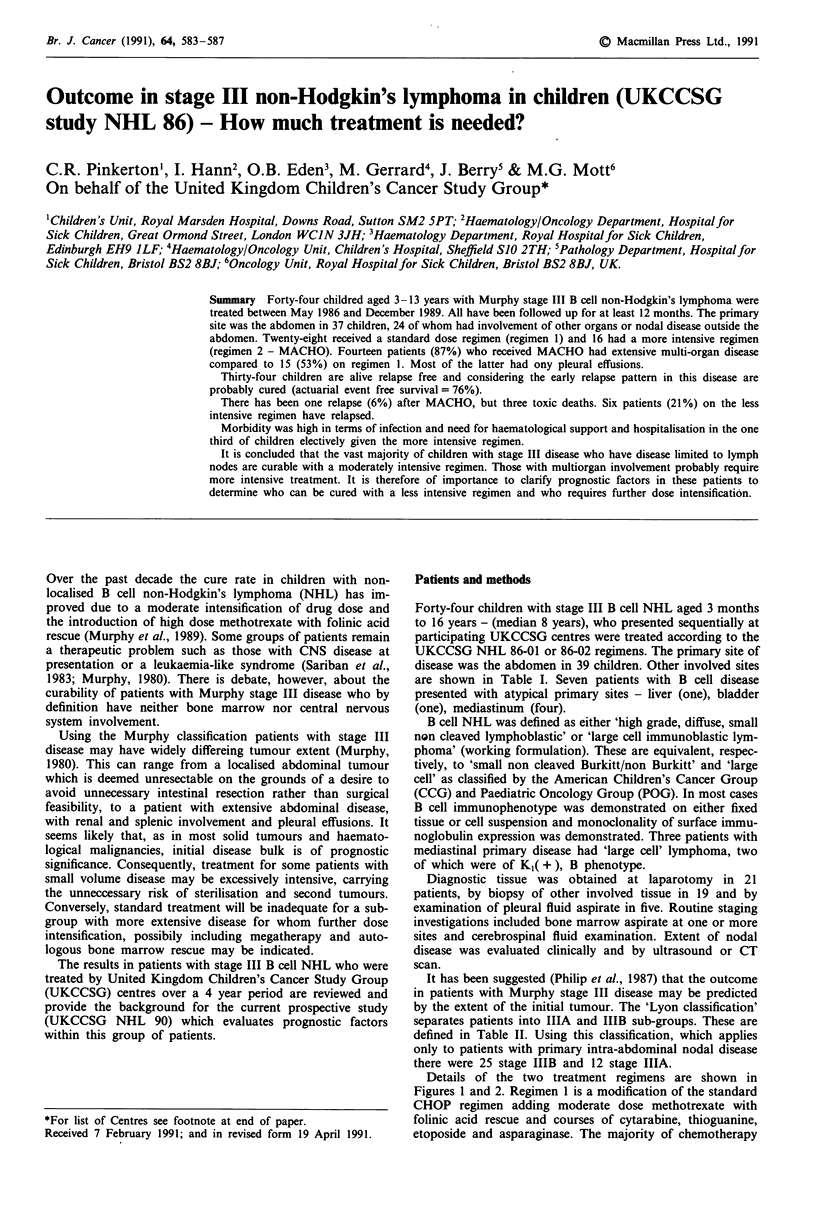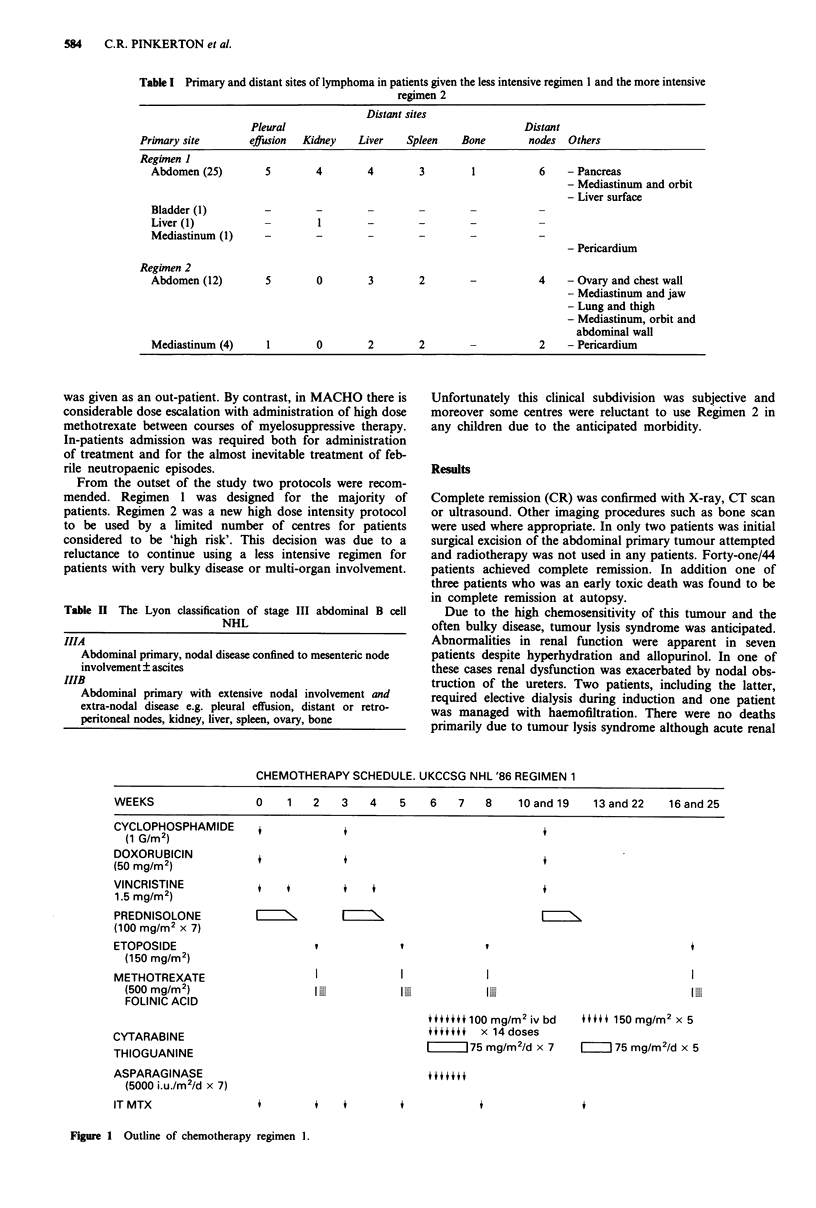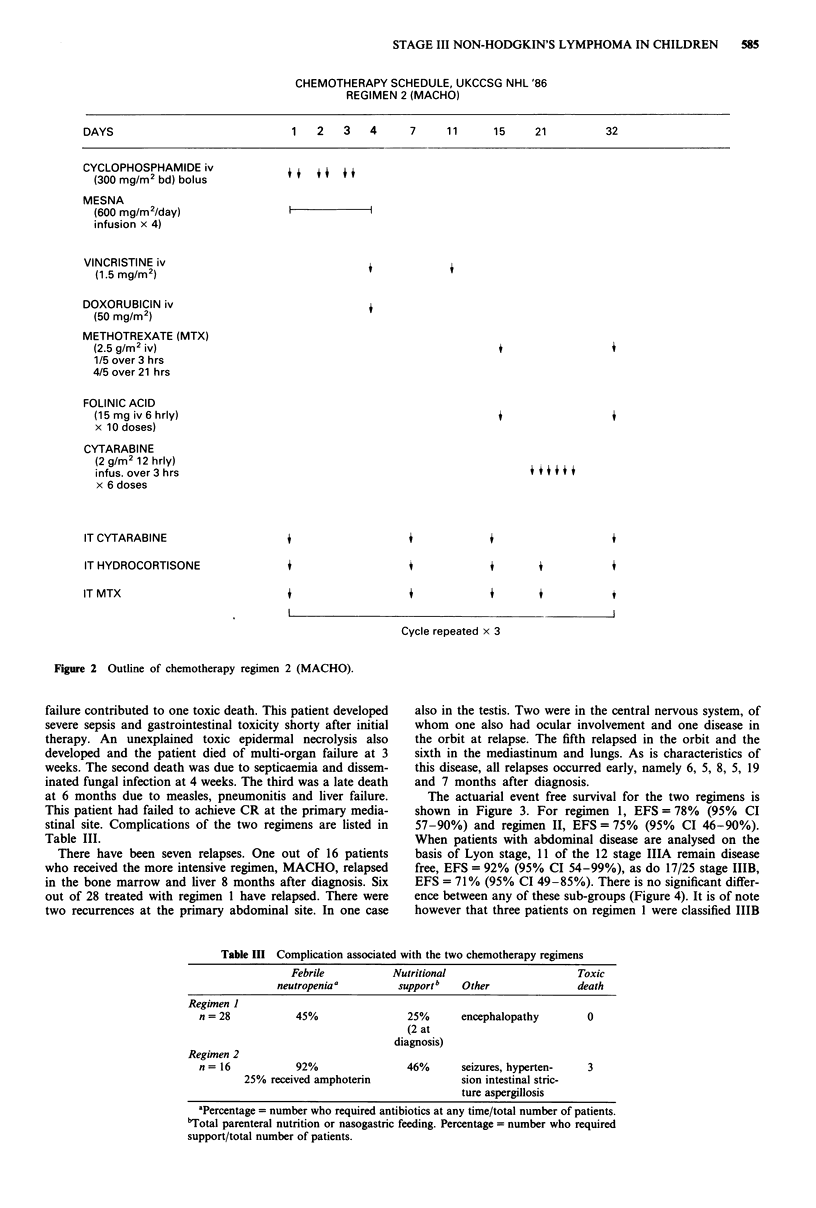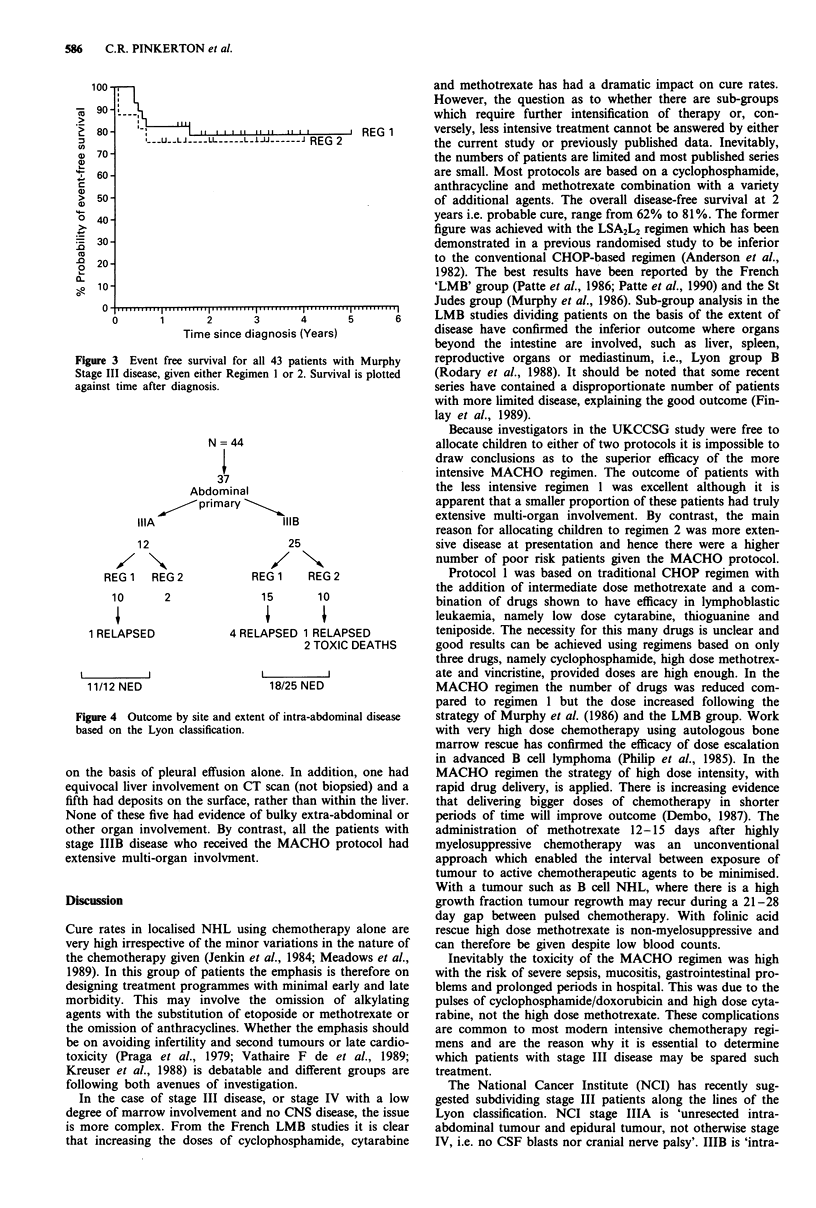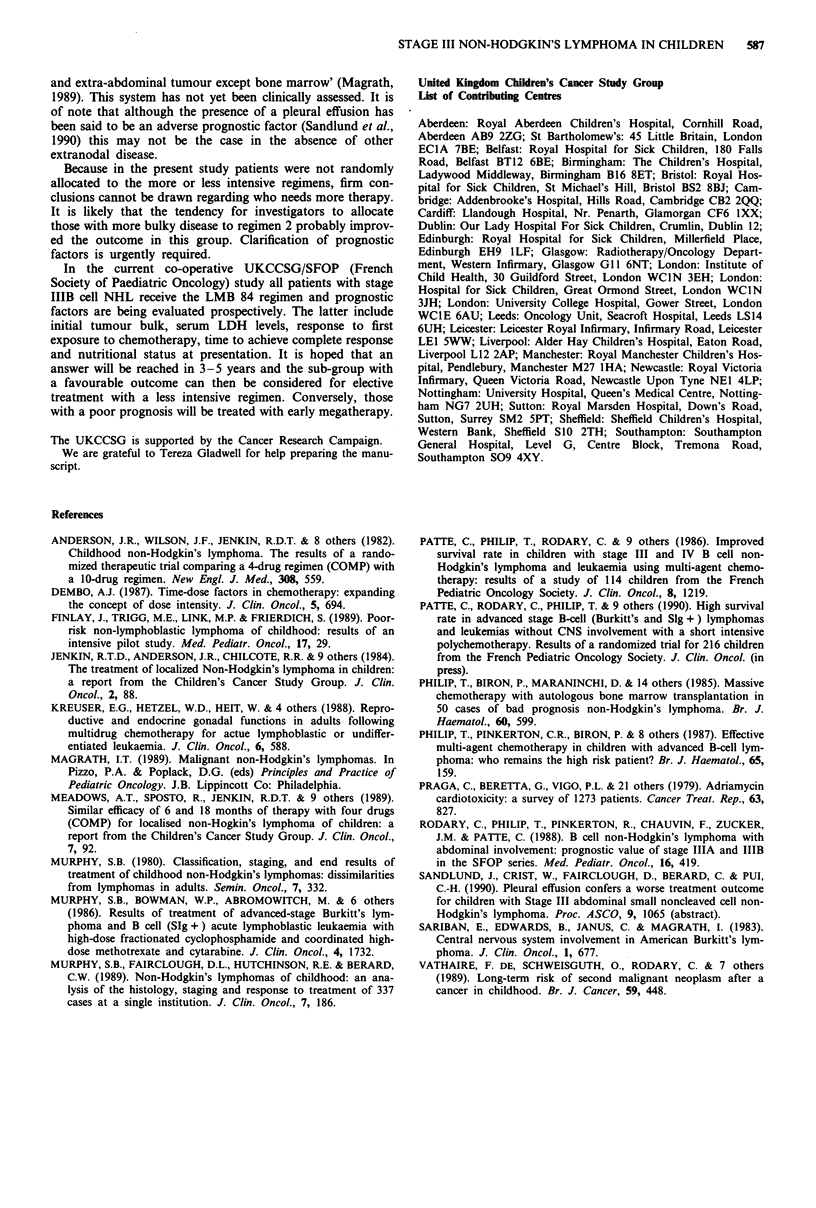# Outcome in stage III non-Hodgkin's lymphoma in children (UKCCSG study NHL 86)--how much treatment is needed? United Kingdom Children's Cancer Study Group.

**DOI:** 10.1038/bjc.1991.354

**Published:** 1991-09

**Authors:** C. R. Pinkerton, I. Hann, O. B. Eden, M. Gerrard, J. Berry, M. G. Mott

**Affiliations:** Children's Unit, Royal Marsden Hospital, Sutton, UK.

## Abstract

Forty-four children aged 3-13 years with Murphy stage III B cell non-Hodgkin's lymphoma were treated between May 1986 and December 1989. All have been followed up for at least 12 months. The primary site was the abdomen in 37 children, 24 of whom had involvement of other organs or nodal disease outside the abdomen. Twenty-eight received a standard dose regimen (regimen 1) and 16 had a more intensive regimen (regimen 2--MACHO). Fourteen patients (87%) who received MACHO had extensive multi-organ disease compared to 15 (53%) on regimen 1. Most of the latter had only pleural effusions. Thirty-four children are alive relapse free and considering the early relapse pattern in this disease are probably cured (actuarial event free survival = 76%). There has been one relapse (6%) after MACHO, but three toxic deaths. Six patients (21%) on the less intensive regimen have relapsed. Morbidity was high in terms of infection and need for haematological support and hospitalisation in the one third of children electively given the more intensive regimen. It is concluded that the vast majority of children with stage III disease who have disease limited to lymph nodes are curable with a moderately intensive regimen. Those with multiorgan involvement probably require more intensive treatment. It is therefore of importance to clarify prognostic factors in these patients to determine who can be cured with a less intensive regimen and who requires further dose intensification.


Br. J. Cancer (1991), 64, 583-587 © Macmillan Press Ltd., 1991

### Outcome in stage III non-Hodgkin's lymphoma in children (UKCCSG study NHL 86) – How much treatment is needed?

C.R. Pinkerton[1], I. Hann[2], O.B. Eden[3], M. Gerrard[4], J. Berry[5] & M.G. Mott[6]
On behalf of the United Kingdom Children's Cancer Study Group*

[1]*Children's Unit, Royal Marsden Hospital, Downs Road, Sutton SM2 5PT;* [2]*Haematology/Oncology Department, Hospital for Sick Children, Great Ormond Street, London WC1N 3JH;* [3]*Haematology Department, Royal Hospital for Sick Children, Edinburgh EH9 1LF;* [4]*Haematology/Oncology Unit, Children's Hospital, Sheffield S10 2TH;* [5]*Pathology Department, Hospital for Sick Children, Bristol BS2 8BJ;* [6]*Oncology Unit, Royal Hospital for Sick Children, Bristol BS2 8BJ, UK.*

**Summary** Forty-four childred aged 3–13 years with Murphy stage III B cell non-Hodgkin's lymphoma were treated between May 1986 and December 1989. All have been followed up for at least 12 months. The primary site was the abdomen in 37 children, 24 of whom had involvement of other organs or nodal disease outside the abdomen. Twenty-eight received a standard dose regimen (regimen 1) and 16 had a more intensive regimen (regimen 2 – MACHO). Fourteen patients (87%) who received MACHO had extensive multi-organ disease compared to 15 (53%) on regimen 1. Most of the latter had ony pleural effusions.

Thirty-four children are alive relapse free and considering the early relapse pattern in this disease are probably cured (actuarial event free survival = 76%).

There has been one relapse (6%) after MACHO, but three toxic deaths. Six patients (21%) on the less intensive regimen have relapsed.

Morbidity was high in terms of infection and need for haematological support and hospitalisation in the one third of children electively given the more intensive regimen.

It is concluded that the vast majority of children with stage III disease who have disease limited to lymph nodes are curable with a moderately intensive regimen. Those with multiorgan involvement probably require more intensive treatment. It is therefore of importance to clarify prognostic factors in these patients to determine who can be cured with a less intensive regimen and who requires further dose intensification.

Over the past decade the cure rate in children with non-localised B cell non-Hodgkin's lymphoma (NHL) has improved due to a moderate intensification of drug dose and the introduction of high dose methotrexate with folinic acid rescue (Murphy *et al.*, 1989). Some groups of patients remain a therapeutic problem such as those with CNS disease at presentation or a leukaemia-like syndrome (Sariban *et al.*, 1983; Murphy, 1980). There is debate, however, about the curability of patients with Murphy stage III disease who by definition have neither bone marrow nor central nervous system involvement.

Using the Murphy classification patients with stage III disease may have widely differeing tumour extent (Murphy, 1980). This can range from a localised abdominal tumour which is deemed unresectable on the grounds of a desire to avoid unnecessary intestinal resection rather than surgical feasibility, to a patient with extensive abdominal disease, with renal and splenic involvement and pleural effusions. It seems likely that, as in most solid tumours and haematological malignancies, initial disease bulk is of prognostic significance. Consequently, treatment for some patients with small volume disease may be excessively intensive, carrying the unneccessary risk of sterilisation and second tumours. Conversely, standard treatment will be inadequate for a sub-group with more extensive disease for whom further dose intensification, possibily including megatherapy and autologous bone marrow rescue may be indicated.

The results in patients with stage III B cell NHL who were treated by United Kingdom Children's Cancer Study Group (UKCCSG) centres over a 4 year period are reviewed and provide the background for the current prospective study (UKCCSG NHL 90) which evaluates prognostic factors within this group of patients.

*For list of Centres see footnote at end of paper.

Received 7 February 1991; and in revised form 19 April 1991.

### Patients and methods

Forty-four children with stage III B cell NHL aged 3 months to 16 years – (median 8 years), who presented sequentially at participating UKCCSG centres were treated according to the UKCCSG NHL 86-01 or 86-02 regimens. The primary site of disease was the abdomen in 39 children. Other involved sites are shown in Table I. Seven patients with B cell disease presented with atypical primary sites – liver (one), bladder (one), mediastinum (four).

B cell NHL was defined as either 'high grade, diffuse, small non cleaved lymphoblastic' or 'large cell immunoblastic lymphoma' (working formulation). These are equivalent, respectively, to 'small non cleaved Burkitt/non Burkitt' and 'large cell' as classified by the American Children's Cancer Group (CCG) and Paediatric Oncology Group (POG). In most cases B cell immunophenotype was demonstrated on either fixed tissue or cell suspension and monoclonality of surface immunoglobulin expression was demonstrated. Three patients with mediastinal primary disease had 'large cell' lymphoma, two of which were of $K_1(+)$, B phenotype.

Diagnostic tissue was obtained at laparotomy in 21 patients, by biopsy of other involved tissue in 19 and by examination of pleural fluid aspirate in five. Routine staging investigations included bone marrow aspirate at one or more sites and cerebrospinal fluid examination. Extent of nodal disease was evaluated clinically and by ultrasound or CT scan.

It has been suggested (Philip *et al.*, 1987) that the outcome in patients with Murphy stage III disease may be predicted by the extent of the initial tumour. The 'Lyon classification' separates patients into IIIA and IIIB sub-groups. These are defined in Table II. Using this classification, which applies only to patients with primary intra-abdominal nodal disease there were 25 stage IIIB and 12 stage IIIA.

Details of the two treatment regimens are shown in Figures 1 and 2. Regimen 1 is a modification of the standard CHOP regimen adding moderate dose methotrexate with folinic acid rescue and courses of cytarabine, thioguanine, etoposide and asparaginase. The majority of chemotherapy

**Table I** Primary and distant sites of lymphoma in patients given the less intensive regimen 1 and the more intensive regimen 2

| | Distant sites | | | | | | |
|---|---|---|---|---|---|---|---|
| *Primary site* | *Pleural effusion* | *Kidney* | *Liver* | *Spleen* | *Bone* | *Distant nodes* | *Others* |
| *Regimen 1* | | | | | | | |
| Abdomen (25) | 5 | 4 | 4 | 3 | 1 | 6 | – Pancreas<br>– Mediastinum and orbit<br>– Liver surface |
| Bladder (1) | – | – | – | – | – | – | |
| Liver (1) | – | 1 | – | – | – | – | |
| Mediastinum (1) | – | – | – | – | – | – | – Pericardium |
| *Regimen 2* | | | | | | | |
| Abdomen (12) | 5 | 0 | 3 | 2 | – | 4 | – Ovary and chest wall<br>– Mediastinum and jaw<br>– Lung and thigh<br>– Mediastinum, orbit and abdominal wall |
| Mediastinum (4) | 1 | 0 | 2 | 2 | – | 2 | – Pericardium |

was given as an out-patient. By contrast, in MACHO there is considerable dose escalation with administration of high dose methotrexate between courses of myelosuppressive therapy. In-patients admission was required both for administration of treatment and for the almost inevitable treatment of febrile neutropaenic episodes.

From the outset of the study two protocols were recommended. Regimen 1 was designed for the majority of patients. Regimen 2 was a new high dose intensity protocol to be used by a limited number of centres for patients considered to be 'high risk'. This decision was due to a reluctance to continue using a less intensive regimen for patients with very bulky disease or multi-organ involvement.

**Table II** The Lyon classification of stage III abdominal B cell NHL

| | |
|---|---|
| *IIIA* | Abdominal primary, nodal disease confined to mesenteric node involvement ± ascites |
| *IIIB* | Abdominal primary with extensive nodal involvement *and* extra-nodal disease e.g. pleural effusion, distant or retroperitoneal nodes, kidney, liver, spleen, ovary, bone |

Unfortunately this clinical subdivision was subjective and moreover some centres were reluctant to use Regimen 2 in any children due to the anticipated morbidity.

### Results

Complete remission (CR) was confirmed with X-ray, CT scan or ultrasound. Other imaging procedures such as bone scan were used where appropriate. In only two patients was initial surgical excision of the abdominal primary tumour attempted and radiotherapy was not used in any patients. Forty-one/44 patients achieved complete remission. In addition one of three patients who was an early toxic death was found to be in complete remission at autopsy.

Due to the high chemosensitivity of this tumour and the often bulky disease, tumour lysis syndrome was anticipated. Abnormalities in renal function were apparent in seven patients despite hyperhydration and allopurinol. In one of these cases renal dysfunction was exacerbated by nodal obstruction of the ureters. Two patients, including the latter, required elective dialysis during induction and one patient was managed with haemofiltration. There were no deaths primarily due to tumour lysis syndrome although acute renal

**Figure 1** Outline of chemotherapy regimen 1.

**Figure 2** Outline of chemotherapy regimen 2 (MACHO).

failure contributed to one toxic death. This patient developed severe sepsis and gastrointestinal toxicity shorty after initial therapy. An unexplained toxic epidermal necrolysis also developed and the patient died of multi-organ failure at 3 weeks. The second death was due to septicaemia and disseminated fungal infection at 4 weeks. The third was a late death at 6 months due to measles, pneumonitis and liver failure. This patient had failed to achieve CR at the primary mediastinal site. Complications of the two regimens are listed in Table III.

There have been seven relapses. One out of 16 patients who received the more intensive regimen, MACHO, relapsed in the bone marrow and liver 8 months after diagnosis. Six out of 28 treated with regimen 1 have relapsed. There were two recurrences at the primary abdominal site. In one case also in the testis. Two were in the central nervous system, of whom one also had ocular involvement and one disease in the orbit at relapse. The fifth relapsed in the orbit and the sixth in the mediastinum and lungs. As is characteristics of this disease, all relapses occurred early, namely 6, 5, 8, 5, 19 and 7 months after diagnosis.

The actuarial event free survival for the two regimens is shown in Figure 3. For regimen 1, EFS = 78% (95% CI 57–90%) and regimen II, EFS = 75% (95% CI 46–90%). When patients with abdominal disease are analysed on the basis of Lyon stage, 11 of the 12 stage IIIA remain disease free, EFS = 92% (95% CI 54–99%), as do 17/25 stage IIIB, EFS = 71% (95% CI 49–85%). There is no significant difference between any of these sub-groups (Figure 4). It is of note however that three patients on regimen 1 were classified IIIB

**Table III** Complication associated with the two chemotherapy regimens

| | *Febrile neutropenia*[a] | *Nutritional support*[b] | *Other* | *Toxic death* |
|---|---|---|---|---|
| *Regimen 1* | | | | |
| $n = 28$ | 45% | 25% (2 at diagnosis) | encephalopathy | 0 |
| *Regimen 2* | | | | |
| $n = 16$ | 92% 25% received amphoterin | 46% | seizures, hypertension intestinal stricture aspergillosis | 3 |

[a]Percentage = number who required antibiotics at any time/total number of patients.
[b]Total parenteral nutrition or nasogastric feeding. Percentage = number who required support/total number of patients.

**Figure 3** Event free survival for all 43 patients with Murphy Stage III disease, given either Regimen 1 or 2. Survival is plotted against time after diagnosis.

**Figure 4** Outcome by site and extent of intra-abdominal disease based on the Lyon classification.

on the basis of pleural effusion alone. In addition, one had equivocal liver involvement on CT scan (not biopsied) and a fifth had deposits on the surface, rather than within the liver. None of these five had evidence of bulky extra-abdominal or other organ involvement. By contrast, all the patients with stage IIIB disease who received the MACHO protocol had extensive multi-organ involvment.

### Discussion

Cure rates in localised NHL using chemotherapy alone are very high irrespective of the minor variations in the nature of the chemotherapy given (Jenkin *et al.*, 1984; Meadows *et al.*, 1989). In this group of patients the emphasis is therefore on designing treatment programmes with minimal early and late morbidity. This may involve the omission of alkylating agents with the substitution of etoposide or methotrexate or the omission of anthracyclines. Whether the emphasis should be on avoiding infertility and second tumours or late cardiotoxicity (Praga *et al.*, 1979; Vathaire F de *et al.*, 1989; Kreuser *et al.*, 1988) is debatable and different groups are following both avenues of investigation.

In the case of stage III disease, or stage IV with a low degree of marrow involvement and no CNS disease, the issue is more complex. From the French LMB studies it is clear that increasing the doses of cyclophosphamide, cytarabine and methotrexate has had a dramatic impact on cure rates. However, the question as to whether there are sub-groups which require further intensification of therapy or, conversely, less intensive treatment cannot be answered by either the current study or previously published data. Inevitably, the numbers of patients are limited and most published series are small. Most protocols are based on a cyclophosphamide, anthracycline and methotrexate combination with a variety of additional agents. The overall disease-free survival at 2 years i.e. probable cure, range from 62% to 81%. The former figure was achieved with the $LSA_2L_2$ regimen which has been demonstrated in a previous randomised study to be inferior to the conventional CHOP-based regimen (Anderson *et al.*, 1982). The best results have been reported by the French 'LMB' group (Patte *et al.*, 1986; Patte *et al.*, 1990) and the St Judes group (Murphy *et al.*, 1986). Sub-group analysis in the LMB studies dividing patients on the basis of the extent of disease have confirmed the inferior outcome where organs beyond the intestine are involved, such as liver, spleen, reproductive organs or mediastinum, i.e., Lyon group B (Rodary *et al.*, 1988). It should be noted that some recent series have contained a disproportionate number of patients with more limited disease, explaining the good outcome (Finlay *et al.*, 1989).

Because investigators in the UKCCSG study were free to allocate children to either of two protocols it is impossible to draw conclusions as to the superior efficacy of the more intensive MACHO regimen. The outcome of patients with the less intensive regimen 1 was excellent although it is apparent that a smaller proportion of these patients had truly extensive multi-organ involvement. By contrast, the main reason for allocating children to regimen 2 was more extensive disease at presentation and hence there were a higher number of poor risk patients given the MACHO protocol.

Protocol 1 was based on traditional CHOP regimen with the addition of intermediate dose methotrexate and a combination of drugs shown to have efficacy in lymphoblastic leukaemia, namely low dose cytarabine, thioguanine and teniposide. The necessity for this many drugs is unclear and good results can be achieved using regimens based on only three drugs, namely cyclophosphamide, high dose methotrexate and vincristine, provided doses are high enough. In the MACHO regimen the number of drugs was reduced compared to regimen 1 but the dose increased following the strategy of Murphy *et al.* (1986) and the LMB group. Work with very high dose chemotherapy using autologous bone marrow rescue has confirmed the efficacy of dose escalation in advanced B cell lymphoma (Philip *et al.*, 1985). In the MACHO regimen the strategy of high dose intensity, with rapid drug delivery, is applied. There is increasing evidence that delivering bigger doses of chemotherapy in shorter periods of time will improve outcome (Dembo, 1987). The administration of methotrexate 12–15 days after highly myelosuppressive chemotherapy was an unconventional approach which enabled the interval between exposure of tumour to active chemotherapeutic agents to be minimised. With a tumour such as B cell NHL, where there is a high growth fraction tumour regrowth may recur during a 21–28 day gap between pulsed chemotherapy. With folinic acid rescue high dose methotrexate is non-myelosuppressive and can therefore be given despite low blood counts.

Inevitably the toxicity of the MACHO regimen was high with the risk of severe sepsis, mucositis, gastrointestinal problems and prolonged periods in hospital. This was due to the pulses of cyclophosphamide/doxorubicin and high dose cytarabine, not the high dose methotrexate. These complications are common to most modern intensive chemotherapy regimens and are the reason why it is essential to determine which patients with stage III disease may be spared such treatment.

The National Cancer Institute (NCI) has recently suggested subdividing stage III patients along the lines of the Lyon classification. NCI stage IIIA is 'unresected intra-abdominal tumour and epidural tumour, not otherwise stage IV, i.e. no CSF blasts nor cranial nerve palsy'. IIIB is 'intraand extra-abdominal tumour except bone marrow' (Magrath, 1989). This system has not yet been clinically assessed. It is of note that although the presence of a pleural effusion has been said to be an adverse prognostic factor (Sandlund *et al.*, 1990) this may not be the case in the absence of other extranodal disease.

Because in the present study patients were not randomly allocated to the more or less intensive regimens, firm conclusions cannot be drawn regarding who needs more therapy. It is likely that the tendency for investigators to allocate those with more bulky disease to regimen 2 probably improved the outcome in this group. Clarification of prognostic factors is urgently required.

In the current co-operative UKCCSG/SFOP (French Society of Paediatric Oncology) study all patients with stage IIIB cell NHL receive the LMB 84 regimen and prognostic factors are being evaluated prospectively. The latter include initial tumour bulk, serum LDH levels, response to first exposure to chemotherapy, time to achieve complete response and nutritional status at presentation. It is hoped that an answer will be reached in 3–5 years and the sub-group with a favourable outcome can then be considered for elective treatment with a less intensive regimen. Conversely, those with a poor prognosis will be treated with early megatherapy.

The UKCCSG is supported by the Cancer Research Campaign.
We are grateful to Tereza Gladwell for help preparing the manuscript.

**United Kingdom Children's Cancer Study Group**
**List of Contributing Centres**

Aberdeen: Royal Aberdeen Children's Hospital, Cornhill Road, Aberdeen AB9 2ZG; St Bartholomew's: 45 Little Britain, London EC1A 7BE; Belfast: Royal Hospital for Sick Children, 180 Falls Road, Belfast BT12 6BE; Birmingham: The Children's Hospital, Ladywood Middleway, Birmingham B16 8ET; Bristol: Royal Hospital for Sick Children, St Michael's Hill, Bristol BS2 8BJ; Cambridge: Addenbrooke's Hospital, Hills Road, Cambridge CB2 2QQ; Cardiff: Llandough Hospital, Nr. Penarth, Glamorgan CF6 1XX; Dublin: Our Lady Hospital For Sick Children, Crumlin, Dublin 12; Edinburgh: Royal Hospital for Sick Children, Millerfield Place, Edinburgh EH9 1LF; Glasgow: Radiotherapy/Oncology Department, Western Infirmary, Glasgow G11 6NT; London: Institute of Child Health, 30 Guildford Street, London WC1N 3EH; London: Hospital for Sick Children, Great Ormond Street, London WC1N 3JH; London: University College Hospital, Gower Street, London WC1E 6AU; Leeds: Oncology Unit, Seacroft Hospital, Leeds LS14 6UH; Leicester: Leicester Royal Infirmary, Infirmary Road, Leicester LE1 5WW; Liverpool: Alder Hay Children's Hospital, Eaton Road, Liverpool L12 2AP; Manchester: Royal Manchester Children's Hospital, Pendlebury, Manchester M27 1HA; Newcastle: Royal Victoria Infirmary, Queen Victoria Road, Newcastle Upon Tyne NE1 4LP; Nottingham: University Hospital, Queen's Medical Centre, Nottingham NG7 2UH; Sutton: Royal Marsden Hospital, Down's Road, Sutton, Surrey SM2 5PT; Sheffield: Sheffield Children's Hospital, Western Bank, Sheffield S10 2TH; Southampton: Southampton General Hospital, Level G, Centre Block, Tremona Road, Southampton SO9 4XY.

### References

ANDERSON, J.R., WILSON, J.F., JENKIN, R.D.T. & 8 others (1982). Childhood non-Hodgkin's lymphoma. The results of a randomized therapeutic trial comparing a 4-drug regimen (COMP) with a 10-drug regimen. *New Engl. J. Med.*, **308**, 559.

DEMBO, A.J. (1987). Time-dose factors in chemotherapy: expanding the concept of dose intensity. *J. Clin. Oncol.*, **5**, 694.

FINLAY, J., TRIGG, M.E., LINK, M.P. & FRIERDICH, S. (1989). Poor-risk non-lymphoblastic lymphoma of childhood: results of an intensive pilot study. *Med. Pediatr. Oncol.*, **17**, 29.

JENKIN, R.T.D., ANDERSON, J.R., CHILCOTE, R.R. & 9 others (1984). The treatment of localized Non-Hodgkin's lymphoma in children: a report from the Children's Cancer Study Group. *J. Clin. Oncol.*, **2**, 88.

KREUSER, E.G., HETZEL, W.D., HEIT, W. & 4 others (1988). Reproductive and endocrine gonadal functions in adults following multidrug chemotherapy for actue lymphoblastic or undifferentiated leukaemia. *J. Clin. Oncol.*, **6**, 588.

MAGRATH, I.T. (1989). Malignant non-Hodgkin's lymphomas. In Pizzo, P.A. & Poplack, D.G. (eds) *Principles and Practice of Pediatric Oncology*. J.B. Lippincott Co: Philadelphia.

MEADOWS, A.T., SPOSTO, R., JENKIN, R.D.T. & 9 others (1989). Similar efficacy of 6 and 18 months of therapy with four drugs (COMP) for localised non-Hogkin's lymphoma of children: a report from the Children's Cancer Study Group. *J. Clin. Oncol.*, **7**, 92.

MURPHY, S.B. (1980). Classification, staging, and end results of treatment of childhood non-Hodgkin's lymphomas: dissimilarities from lymphomas in adults. *Semin. Oncol.*, **7**, 332.

MURPHY, S.B., BOWMAN, W.P., ABROMOWITCH, M. & 6 others (1986). Results of treatment of advanced-stage Burkitt's lymphoma and B cell (SIg+) acute lymphoblastic leukaemia with high-dose fractionated cyclophosphamide and coordinated high-dose methotrexate and cytarabine. *J. Clin. Oncol.*, **4**, 1732.

MURPHY, S.B., FAIRCLOUGH, D.L., HUTCHINSON, R.E. & BERARD, C.W. (1989). Non-Hodgkin's lymphomas of childhood: an analysis of the histology, staging and response to treatment of 337 cases at a single institution. *J. Clin. Oncol.*, **7**, 186.

PATTE, C., PHILIP, T., RODARY, C. & 9 others (1986). Improved survival rate in children with stage III and IV B cell non-Hodgkin's lymphoma and leukaemia using multi-agent chemotherapy: results of a study of 114 children from the French Pediatric Oncology Society. *J. Clin. Oncol.*, **8**, 1219.

PATTE, C., RODARY, C., PHILIP, T. & 9 others (1990). High survival rate in advanced stage B-cell (Burkitt's and Slg+) lymphomas and leukemias without CNS involvement with a short intensive polychemotherapy. Results of a randomized trial for 216 children from the French Pediatric Oncology Society. *J. Clin. Oncol.* (in press).

PHILIP, T., BIRON, P., MARANINCHI, D. & 14 others (1985). Massive chemotherapy with autologous bone marrow transplantation in 50 cases of bad prognosis non-Hodgkin's lymphoma. *Br. J. Haematol.*, **60**, 599.

PHILIP, T., PINKERTON, C.R., BIRON, P. & 8 others (1987). Effective multi-agent chemotherapy in children with advanced B-cell lymphoma: who remains the high risk patient? *Br. J. Haematol.*, **65**, 159.

PRAGA, C., BERETTA, G., VIGO, P.L. & 21 others (1979). Adriamycin cardiotoxicity: a survey of 1273 patients. *Cancer Treat. Rep.*, **63**, 827.

RODARY, C., PHILIP, T., PINKERTON, R., CHAUVIN, F., ZUCKER, J.M. & PATTE, C. (1988). B cell non-Hodgkin's lymphoma with abdominal involvement: prognostic value of stage IIIA and IIIB in the SFOP series. *Med. Pediatr. Oncol.*, **16**, 419.

SANDLUND, J., CRIST, W., FAIRCLOUGH, D., BERARD, C. & PUI, C.-H. (1990). Pleural effusion confers a worse treatment outcome for children with Stage III abdominal small noncleaved cell non-Hodgkin's lymphoma. *Proc. ASCO*, **9**, 1065 (abstract).

SARIBAN, E., EDWARDS, B., JANUS, C. & MAGRATH, I. (1983). Central nervous system involvement in American Burkitt's lymphoma. *J. Clin. Oncol.*, **1**, 677.

VATHAIRE, F. DE, SCHWEISGUTH, O., RODARY, C. & 7 others (1989). Long-term risk of second malignant neoplasm after a cancer in childhood. *Br. J. Cancer*, **59**, 448.